# Clinical decision-making on spinal cord injury-associated pneumonia: a nationwide survey in Germany

**DOI:** 10.1038/s41393-020-0435-5

**Published:** 2020-02-18

**Authors:** Claudia Druschel, Ramin R. Ossami Saidy, Ulrike Grittner, Claus P. Nowak, Andreas Meisel, Klaus-Dieter Schaser, Andreas Niedeggen, Thomas Liebscher, Marcel A. Kopp, Jan M. Schwab

**Affiliations:** 1 Department of Neurology with Experimental Neurology, Charité-Universitätsmedizin Berlin, Corporate Member of Freie Universität Berlin, Humboldt-Universität zu Berlin, and Berlin Institute of Health, Charitéplatz 1, 10117 Berlin, Germany; 2 Clinical and Experimental Spinal Cord Injury Research (Neuroparaplegiology), Charité-Universitätsmedizin Berlin, Berlin, Germany; 3 Department of Orthopaedic and Trauma Surgery, Universitätsklinikum Carl-Gustav Carus, Dresden, Germany; 4 Department of Surgery, Charité-Universitätsmedizin Berlin, Berlin, Germany; 5 Institute of Biometry and Clinical Epidemiology, Charité-Universitätsmedizin Berlin, Berlin, Germany; 6 Berlin Institute of Health (BIH), Berlin, Germany; 7 NeuroCure Clinical Research Center, Charité-Universitätsmedizin Berlin, Berlin, Germany; 8 Center for Stroke Research Berlin, Charité-Universitätsmedizin Berlin, Berlin, Germany; 9 Treatment Centre for Spinal Cord Injuries, Trauma Hospital Berlin, Berlin, Germany; 10 Berlin Institute of Health, QUEST-Center for Transforming Biomedical Research, Berlin, Germany; 11 Department of Neurology, Spinal Cord Medicine (Paraplegiology), The Neurological Institute, The Ohio State University, Wexner Medical Center, Columbus, OH USA; 12 Belford Center for Spinal Cord Injury, Departments of Neuroscience and Physical Medicine and Rehabilitation, The Neurological Institute, The Ohio State University, Wexner Medical Center, Columbus, OH USA

**Keywords:** Respiratory signs and symptoms, Diagnostic markers, Diagnosis, Spinal cord diseases, Bacterial infection

## Abstract

**Study design:**

Survey study.

**Objectives:**

Spinal cord injury (SCI)-associated pneumonia (SCI-AP) is associated with poor functional recovery and a major cause of death after SCI. Better tackling SCI-AP requires a common understanding on how SCI-AP is defined. This survey examines clinical algorithms relevant for diagnosis and treatment of SCI-AP.

**Setting:**

All departments for SCI-care in Germany.

**Methods:**

The clinical decision-making on SCI-AP and the utility of the Centers for Disease Control and Prevention (CDC) criteria for diagnosis of ‘clinically defined pneumonia’ were assessed by means of a standardized questionnaire including eight case vignettes of suspected SCI-AP. The diagnostic decisions based on the case information were analysed using classification and regression trees (CART).

**Results:**

The majority of responding departments were aware of the CDC-criteria (88%). In the clinical vignettes, 38–81% of the departments diagnosed SCI-AP in accordance with the CDC-criteria and 7–41% diagnosed SCI-AP in deviation from the CDC-criteria. The diagnostic agreement was not associated with the availability of standard operating procedures for SCI-AP management in the departments. CART analysis identified radiological findings, fever, and worsened gas exchange as most important for the decision on SCI-AP. Frequently requested supplementary diagnostics were microbiological analyses, C-reactive protein, and procalcitonin. For empirical antibiotic therapy, the departments used (acyl-)aminopenicillins/β-lactamase inhibitors, cephalosporins, or combinations of (acyl-)aminopenicillins/β-lactamase inhibitors with fluoroquinolones or carbapenems.

**Conclusions:**

This survey reveals a diagnostic ambiguity regarding SCI-AP despite the awareness of CDC-criteria and established SOPs. Heterogeneous clinical practice is encouraging the development of disease-specific guidelines for diagnosis and management of SCI-AP.

## Introduction

Spinal cord injury (SCI)-associated pneumonia (SCI-AP) is a very frequent complication [1] and a leading cause of death after SCI [2–4]. Moreover, SCI-AP acquired during acute and inpatient rehabilitative care is an outcome-modifying factor associated with poor neurological and functional long-term recovery [5–7].

A sensitive, specific, and feasible diagnostic pathway is essential for decision-making in the management of SCI-AP. The Centers for Disease Control and Prevention (CDC) has provided criteria for ‘clinically defined pneumonia’ established and verified for the definition of healthcare (HAP)- and community (CAP)-associated pneumonia [8]. However, current standards for disease-specific acute management of SCI have not yet implemented such pathways [9, 10]. Moreover, the pathophysiology of SCI-AP is complex. Independently of risk factors such as mechanical ventilation or loss of the expiratory intercostal and abdominal muscle function with subsequent decrease of vital capacity and cough [11], the increased susceptibility to SCI-AP is additionally facilitated by the Spinal Cord Injury-induced Immune Deficiency Syndrome (SCI-IDS) [12–14], characterized through a neuroanatomical lesion-height dependent decrease in antibody synthesis and cellular immune-response leading to impaired host defence [15–17]. In the context of the SCI-IDS, some diagnostic CDC-criteria for a ‘clinically defined pneumonia’, e.g. leucocyte counts, are substantially altered due to SCI itself [13, 14] and may therefore be of limited significance for the diagnosis of SCI-AP.

In order to define a starting point for the development of SCI-specific guidance for the diagnosis of SCI-AP, this survey investigates the clinical algorithms relevant for SCI-AP and analyses the utility of the CDC-criteria for the diagnosis of SCI-AP and decision-making on its therapy.

## Methods

### Survey

A standardized questionnaire was sent to the German intensive care units, trauma-, orthopaedic- and neurosurgery departments in May 2013 (see Supplementary Appendix for an English translation of the survey questionnaire). The departments were selected from university hospitals and specialized non-university SCI-care centres organized within the German-speaking Society for Paraplegia (DMGP). Departments which had indicated not to treat people with SCI in a recent previous nationwide SCI-survey [18] conducted by the study group were excluded. University-affiliated teaching hospitals located off-campus were also withdrawn from the study sample as they had indicated only a “sporadic” treatment of individuals with SCI (<10/year) in the previous survey and thus were not representing departments with established SCI-care algorithms. The survey was addressed to the medical directors of the departments hosting responsibility for the clinical practice in their centres. The medical directors filled the questionnaire personally, which in most cases included consultations with the respective physician team of the department. All returned questionnaires were checked for misinterpretations or indistinct answers before they were processed for statistical analysis.

### Questionnaire

**Fig. 1:**
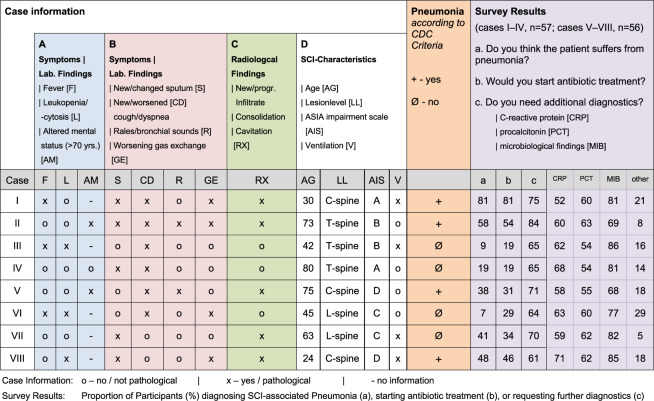
Synopsis of case vignettes and survey results. Each of the eight cases presents a pattern of clinical symptoms, laboratory values, radiological findings, and SCI characteristics (Blocks A–D). The cases were designed to either diagnose or exclude pneumonia (Block ‘Pneumonia', orange). According to CDC-criteria, at least one positive finding in Block A and with at least two positive findings in Block B in combination with one positive finding in Block C (exception: pre-existing pulmonary disease is requiring at least two repeated positive chest X-ray findings) allow for diagnosis of pneumonia. Overview of participants’ answers (relative frequencies in percent) to each of the three questions per case (Block ‘Survey Results in %’, purple).

The questionnaire was modelled in analogy to a prior survey on stroke-associated pneumonia [19] and consisted of three sections. Section one assessed care provider profiles, i.e. numbers of treated SCI cases per year, department size, existence of standard operating procedures (SOP) for SCI-AP. Section two provided eight case vignettes of acute traumatic SCI in adult individuals specifying common clinical situations with varying information on parameters relevant for diagnosis of ‘clinically defined pneumonia’ according to CDC-criteria (also referred to as PNU1) [8]. The diagnostic algorithm for ‘clinically defined pneumonia’ comprises combinations of the following timely available clinical and laboratory parameters: leukopenia or leucocytosis, altered mental status in patients ≥70 years of age, fever >38 °C, new onset or changes in purulent sputum or respiratory secretions, new onset of cough/dyspnoea, pathological auscultatory findings, worsening gas exchange such as O_2_ desaturations, increased O_2_ requirements, or increased ventilator demand, tachypnea, and new or progressive infiltrations, consolations or cavitation in chest X-ray [8]. In addition to the CDC-criteria, information on age, comorbidities, mechanical ventilation, neurological level of SCI, and the injury completeness as classified by the ASIA Impairment Scale (AIS) was provided in the case vignettes (Fig. 1). The clinicians were asked to decide (a) whether the patient suffered from SCI-AP, (b) if antibiotic treatment should be initiated, and (c) if further parameters were needed to decide on the diagnosis of SCI-AP. Section three examined the type of antibiotic treatment, its duration, and monitoring as well as the use of prophylactic antibiotics, and prevention of aspiration/dysphagia. The questionnaire was designed by two authors (CD, JMS). In order to carve out putative misunderstandings or inconsistencies, and evaluate the acceptance, four authors representing specialist physicians and/or clinical scientists experienced in stroke or SCI-care and research (AM, AN, TL, MAK) were assigned the task.

### Statistical analysis

Summary descriptive statistics were reported as absolute or relative frequencies. Odds ratios (OR) and 95% confidence intervals (95% CI) were calculated to explore effects of SOPs on diagnosis and management of SCI-AP. These analyses were performed using the software GraphPad Prism, Version 5.0. The classification and regression trees (CART) method was used to investigate the relevance of specific parameters in diagnostic decision-making [20]. All information provided by the case vignettes (Fig. 1), particularly all CDC-criteria applicable for diagnosis of ‘clinically defined pneumonia’, were included in the model using the target variable ‘diagnosis of SCI-AP’. In addition, the department was included as a random effect to account for the individual departments’ tendency to diagnose SCI-AP for different case vignettes. As a sensitivity analysis the regression tree was calculated first with and second without the random effect for the individual department. CART analysis splits the data towards an increase in purity of the subsequent nodes considering the contribution of the input variables to the classification. For description of the increase in purity of the nodes, the relative and absolute increase in the number of correctly classified cases were indicated. In addition, we calculated an improvement score characterizing the decrease of impurity of the nodes based on the Gini Index [21]. CART analysis was performed using the software ‘R’, packages ‘rpart’, Version 4.1–11 [22] and ‘REEMtree’, Version 0.90.3 [23].

## Results

### Survey response

In total, 61 of 161 addressed departments (38%) responded to the questionnaire (Table 1). Fifty-seven of the 61 responding departments were treating individuals with SCI and the majority of them were having more than 10 beds (79%) and used SOP for the management of SCI-AP (66%, Table 2). Most frequent types of infections occurring during the inpatient treatment of SCI in the responding departments were pulmonary (47%), urological (25%), and gastro-intestinal (13%). Awareness of diagnostic CDC-criteria for pneumonia was confirmed by 49 of 56 responding departments for SCI-care (88%, 1 missing). Of the 49 departments aware of the CDC-criteria, 14 (29%) considered them highly relevant, 34 (69%) moderately relevant, and 1 (2%) non-relevant.

**Table 1:** Responder ratio.

Category of department	Addressed	Responded
All hospitals, *n* (%)	161 (100)	61 (37.9)
Intensive care units, *n* (%)	49 (100)	18 (36.7)
Trauma/orthopaedic surgery, *n* (%)	55 (100)	16 (29.1)
Neurosurgery, *n* (%)	32 (100)	15 (46.8)
DMGP, *n* (%)	25 (100)	12 (48.0)

Type of departments that were addressed and that have responded. *DMGP* Deutschsprachige Medizinische Gesellschaft für Paraplegiologie (German Speaking Medical Society for Paraplegiology).

**Table 2:** Profiles of responding departments.

SCI patients treated per year (*n* = 61)	
None, *n* (%)	4 (6.6)
≤10, *n* (%)	14 (23.0)
11–40, *n* (%)	24 (39.3)
>40, *n* (%)	19 (31.1)
Beds for SCI-care (*n* = 57; *n* = 4 without SCI-care excluded)	
≤10, *n* (%)	12 (21.1)
>10, *n* (%)	45 (78.9)
SOP for SCI-AP (*n* = 56, *n* = 4 without SCI-care excluded, *n* = 1 missing)	
Yes, *n* (%)	37 (66.1)
No, *n* (%)	19 (33.9)

*SCI-AP* spinal cord injury-associated pneumonia, *SOP* standard operating procedure.

### Decisions based on case vignettes

The eight case vignettes designed to evaluate the agreement between clinical judgement on SCI-AP and the definition of ‘clinically defined pneumonia’ based on CDC-criteria were rated by the 57 responding departments treating SCI (Fig. 1).

The accuracy in decision-making on SCI-AP in accordance with CDC-criteria was highly varying between the case vignettes. In cases fulfilling the CDC-criteria, a decision on SCI-AP was made by 46 of 57 departments in case I (81%), by 33 of 57 departments in case II (58%), by 21 of 56 departments in case V (38%, 1 missing), and by 27 of 56 departments in case VIII (48%, 1 missing). In the case vignettes not fulfilling the CDC-criteria, a diagnosis of SCI-AP was made by 5 of 57 departments in case III (9%), by 11 of 57 departments in case IV (19%), by 4 of 56 departments in case VI (7%, 1 missing), and by 23 of 56 departments in case VII (41%, 1 missing).

The decision to initiate an antibiotic therapy was largely overlapping with the decision on the diagnosis of SCI-AP and it was ranging from 38% to 81% in the cases with CDC-conform decisions on SCI-AP and from 9–41% in the cases not fulfilling CDC-definitions (Fig. 1).

The vast majority of departments (61–84%) stated to require additional diagnostic parameters for the diagnosis of SCI-AP. The most frequently demanded supplementary diagnostics were microbiological testing (68–86%), C-reactive protein (53–71%), and procalcitonin (54–61%), regardless of the diagnostic or therapeutic decisions made in the individual case vignette (Fig.1). In addition, 5–29% of the departments asked for other supplementary diagnostics, such as ‘additional computer tomography’ (19%), ‘bronchoscopy’ (11%), search for ‘other infections’ (13%), or ‘urinary findings’ (22%). Notably, ‘urinary findings’ were requested most frequently in case VI (47%) that strongly suggested an infection without clinical signs of pneumonia (Fig. 1).

### Parameters relevant for decision-making

**Fig. 2:**
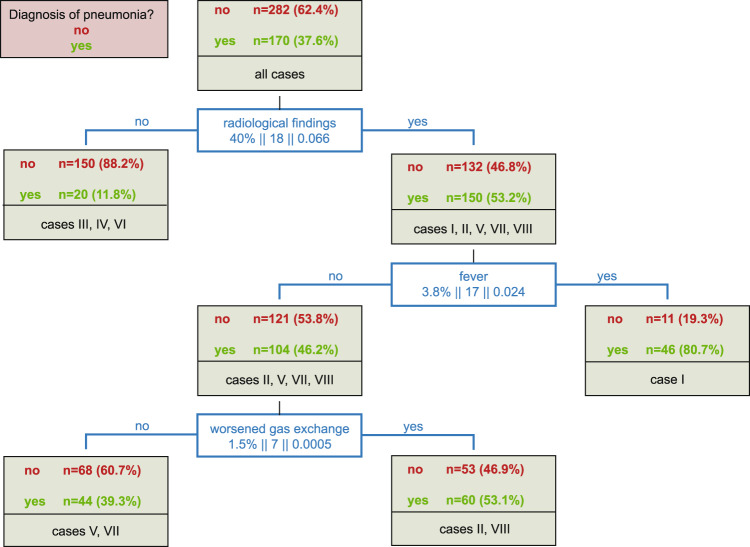
Classification and regression tree (CART) analysis of criteria in decision-making on SCI-associated pneumonia. The regression tree classified 452 ratings, where 57 clinicians were asked to diagnose the same eight case vignettes. Within the bins of the decision tree (blue boxes), the criteria relevant for the diagnosis are indicated together with the related percentage in improvement in accuracy regarding correctly classified cases, the absolute increase in correctly classified cases, and the improvement score indicating the decrease in impurity of the terminal nodes.

The CART analysis based on the eight case vignettes revealed that among the CDC-criteria ‘radiological findings’, ‘fever’, and ‘worsened gas exchange’ were most relevant for decision-making on SCI-AP (Fig. 2). In total, 452 of 456 returned case vignettes were completely filled and thus eligible for statistical evaluation (8 vignettes, 57 departments). For the three vignettes without pathological X-ray findings, 150 of 170 ratings (88%) have not diagnosed SCI-AP. In case of suggested pathologies in X-ray (5 vignettes × 57 departments, 282 ratings, 3 missing), fever was used next to refine the diagnosis. In one vignette with radiological findings and fever SCI-AP was diagnosed in 46 of 57 ratings (81%). However, if fever was not reported (4 vignettes × 57 departments, 225 ratings, 3 missing), the departments decided against the diagnosis of SCI-AP in 121 of 225 ratings (54%). If radiological investigations implied pathology and neither fever nor worsened gas exchange were reported (2 vignettes × 57 departments, 112 ratings, 2 missing), still 44 of 112 (39%) ratings diagnosed SCI-AP.

Except fever, clinical signs such as pathological auscultation findings, worsened cough, or dyspnoea had no influence on decision-making in this model, neither had the patients’ ventilator status. Furthermore, inclusion of the individual department as a random effect in the CART analysis had no influence on the results in this study, implying that there was no strong individual tendency of the department towards an over- or under-diagnosis of SCI-AP.

### Influence of SOPs

Estimating the effects of internal SOPs for the management of SCI-AP in the departments, the availability of SOP had neither an effect on the diagnosis of SCI-AP nor on the request of further diagnostic parameters as indicated by ORs fluctuating around 1.0. Remarkably, the ORs for the effect of SOP on the initiation of an empirical antibiotic treatment are for each case vignette smaller than 1.0. This is indicating a tendency of the centres with SOP to be more restrictive in starting antibiotic treatment (Table 3). The existence of SOP was not clearly associated with the duration of the antibiotic treatment, its follow-up control, or the decision for a prophylactic use of antibiotics (Table 4).

**Table 3:** Potential effects of SOP on diagnosis of SCI-AP, antibiotic treatment decision, and request of further diagnostic tests based on the eight case vignettes.

Case	Diagnosis of SCI-AP			Treatment of SCI-AP			Further diagnostics		
	SOP+,*n* (%)	SOP−, *n* (%)	Odds ratio (95% CI)	SOP+, *n* (%)	SOP−, *n* (%)	Odds ratio (95% CI)	SOP+, *n* (%)	SOP− *n* (%)	Odds ratio (95% CI)
I	28 (78.0)	16 (84.2)	0.65 (0.15–2.83)	28 (78.0)	16 (84.2)	0.66 (0.15–2.83)	29 (80.5)	13 (68.4)	1.91 (0.54–6.82)
II	18 (50.0)	14 (73.7)	0.35 (0.11–1.20)	17 (47.2)	13 (68.4)	0.41 (0.13–1.32)	32 (88.9)	15 (78.9)	2.13 (0.47–9.71)
III	4 (11.0)	1 (5.3)	2.25 (0.23–21.71)	7 (19.4)	4 (21.1)	0.91 (0.23–3.59)	22 (61.1)	15 (78.9)	0.42 (0.12–1.52)
IV	5 (14.0)	6 (31.6)	0.34 (0.09–1.35)	6 (16.7)	5 (26.3)	0.56 (0.15–2.15)	22 (61.1)	14 (73.7)	0.56 (0.17–1.90)
V	11 (31.0)	10 (52.6)	0.40 (0.13–1.25)	10 (27.8)	7 (36.8)	0.66 (0.20–2.15)	27 (75.0)	12 (63.2)	1.75 (0.53–5.81)
VI	2 (6.0)	2 (10.5)	0.50 (0.65–3.86)	8 (22.3)	8 (42.1)	0.39 (0.12–1.31)	23 (63.9)	12 (63.2)	1.03 (0.32–3.27)
VII	16 (44.0)	7 (36.8)	1.37 (0.44–4.29)	11 (33.4)	7 (36.8)	0.75 (0.23–2.43)	27 (75.0)	12 (63.2)	1.75 (0.53–5.08)
VIII	18 (50.0)	9 (47.4)	1.11 (0.37–3.38)	17 (47.2)	9 (47.4)	0.99 (0.33–3.03)	23 (63.9)	11 (57.9)	1.29 (0.41–4.01)

The effect of available SOP (SOP+) vs. unavailable SOP (SOP−) is described by odds ratios. Odds ratios <1.0 indicate a negative association with available SOP. *SCI-AP* spinal cord injury-associated pneumonia, *SOP* standard operating procedure.

**Table 4:** Influence of SOP on antibiotic treatment of SCI-AP.

Antibiotic treatment	SOP+, *n* (%)	SOP−, *n* (%)	Odds ratio (95% CI)
	*n* **=** 36	*n* = 19	
Fixed treatment duration	26 (72.2)	14 (73.7)	0.93 (0.26–3.26)
Antibiotic prophylaxis	5 (13.9)	1 (5.3)	2.90 (0.31–26.86)
Treatment control			
Clinical	32 (88.9)	19 (100)	0.19 (0.01–3.63)
Laboratory	35 (97.2)	19 (100)	0.61 (0.02–15.6)
Chest X-ray	26 (72.2)	17 (89.5)	0.31 (0.06–1.57)

*SCI-AP* spinal cord injury-associated pneumonia, *SOP* standard operating procedure.

### Empirical antibiotic treatment

**Fig. 3:**
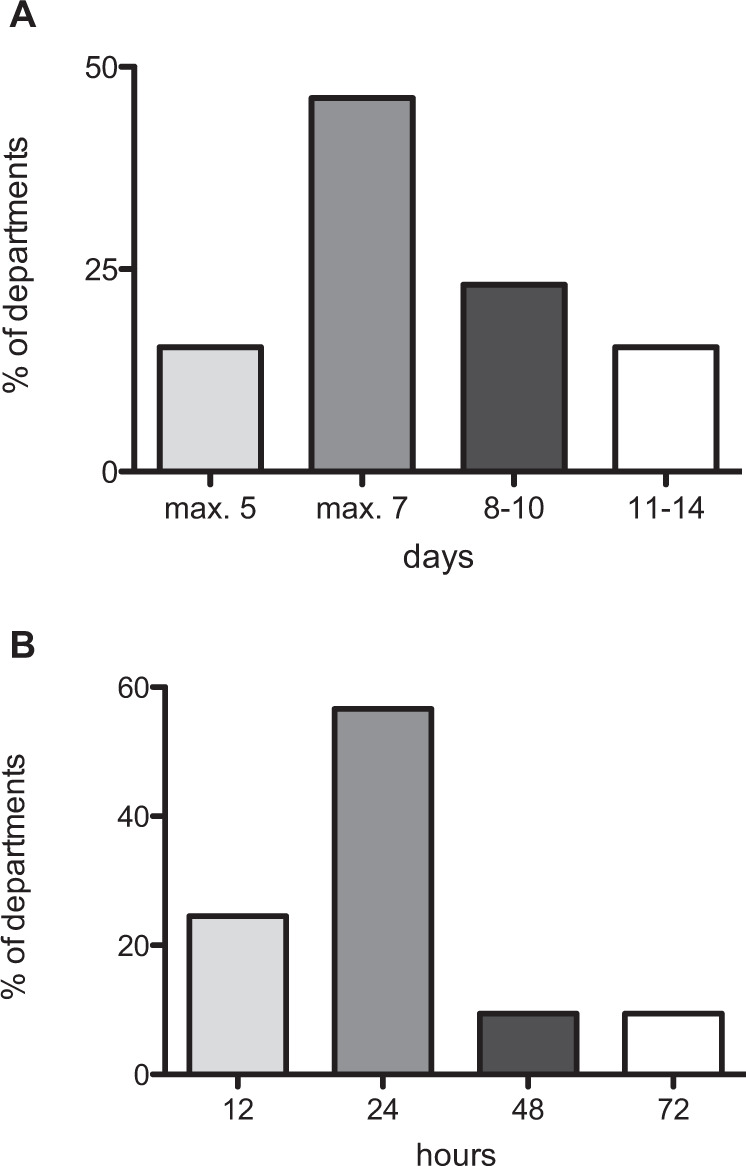
Antibiotic treatment duration and first monitoring of therapeutic success. **a** Duration of antibiotic treatment after diagnosis of SCI-associated pneumonia. **b** Control of the therapeutic success after first antibiotic regimen initiation. SCI spinal cord injury.

All departments for SCI-care (*n* = 57) applied the empirical antibiotic monotherapy of SCI-AP intravenously. In the departments several substances were used in parallel for monotherapy, whereby (acyl-)aminopenicillins/β-lactamase inhibitors were used in 49 of the departments (86%) followed by group 3 cephalosporins, and fluoroquinolones in 17 of the departments each (30%). Further antibiotics for monotherapy were aminopenicillins/β-lactamase inhibitors in 12 (21%) and group 2 cephalosporins in 10 of the departments (18%). Antibiotic combination therapy was reported by 29 of 53 departments (54%, 4 missing) applying prevailingly fluoroquinolones combined with either acyl-aminopenicillins/β-lactamase inhibitors or carbapenems. In the majority of departments (*n* = 42, 74%) the treatment duration was ranging from up to 5 days to 2 weeks (Fig. 3a).

All departments (*n* = 57) stated to start monitoring the therapeutic success mainly within 24 h after the initiation of treatment by clinical examinations in 53 departments (93%), laboratory tests in 56 departments (98%), or radiological means in 44 departments (77%) (Fig. 3b). The vast majority of centres (*n* = 51, 90%) did not use antibiotic prophylaxis. All departments performed physical respiratory therapy as soon as possible after SCI and 46 (82%) applied prophylaxis of dysphagia/aspiration.

## Discussion

As a major result, the survey revealed that the clinical decision-making on SCI-AP in Germany is inconsistent across specialized centres for SCI-care, when compared with the CDC-criteria for ‘clinically defined pneumonia’ as a reference. The 38–81% agreement of the departments’ clinical judgement with the CDC-criteria for ‘clinically defined pneumonia’ in this study is below the inter-observer agreement of 82% correctly diagnosed HAP in a population of critically ill patients in the Netherlands [24]. This can generally be explained by different classification systems, as quantified in a meta-analysis of stroke studies [25]. However, under more complex conditions of mechanical ventilation, the correct diagnosis of ventilator-associated pneumonia (VAP) is more difficult to reach even within the system of CDC-criteria, as revealed in the Netherlands study, where only 68% of the observers agreed on VAP-diagnose [24]. The fact that in this survey 88% of the departments for SCI-care stated to be aware of the CDC-criteria but only 29% ranked them of high relevance pinpoints their limited conclusiveness or feasibility for diagnosis of SCI-AP. Not exclusively, but particularly in cases of elderly people with altered mental status (cases II and V) or in cases without fever and with few specific symptoms (cases VII and VIII) the clinical decisions were ambiguous.

The main clinical items relevant for the diagnosis of SCI-AP revealed in the CART analysis were chest X-ray, fever, and worsened gas exchange. Radiological findings as a central aspect of CDC-criteria are a general recommendation for diagnosis of HAP as confirmed also for SCI-AP by this survey. However, chest X-ray may be inconclusive in bedridden patients [26]. This is probably one of the reasons why there is an apparent heterogeneity in the diagnosis of SCI-AP in this study, independently of the departments’ specialization and its individual tendency to find a diagnostic decision. By comparison with other insults to the CNS, a minor importance of chest X-ray in a survey study on stroke-associated pneumonia [19] may be explained by different characteristics of distinct patient populations. Higher rates of elderly stroke patients with underlying cardiac and/or pulmonary diseases [27, 28] are complicating the interpretation of the chest X-ray and thus limit its diagnostic value. Consequently, the stroke consensus group-modified diagnostic CDC recommendations for stroke-associated pneumonia [29].

The very frequent request for inflammatory markers in terms of C-reactive protein and procalcitonin in this study is supporting the importance of these parameters for clinical judgement. However, because consistent evidence for the specificity of C-reactive protein and procalcitonin for the diagnosis of ‘clinically defined pneumonia’ is lacking [29–31], both parameters cannot currently be recommended as diagnostic criteria. In addition, microbiological findings were also frequently requested as they are part of the CDC-criteria for ‘pneumonia with common bacterial or viral pathogens and specific lab findings’ (referred to as PNU2 in the CDC-definitions) [8]. Although microbiological findings are indispensable for the choice of specific antibiotics, we have focused on timely available criteria for ‘clinically defined pneumonia’ as they allow for rapid decisions on empirical antibiotics. Forthcoming diagnostic algorithms of SCI-AP can benefit from a categorization of individuals with SCI as being immune compromised by incorporating clinical and specific laboratory indicators of the SCI-IDS [7, 15, 17, 32].

In this study, SOPs established in the departments have only minimal impact on the management of SCI-AP. Although centres with available internal SOP tend to be more restrictive with the start of antibiotic treatment, large differences in diagnosis, treatment, and control of SCI-AP between the departments with and without SOP were not evident. Establishing consistent pathways in diagnosis and management of SCI-AP is a prerequisite for improving the quality of SCI-care, since adherence to SOPs has been demonstrated by others to be associated with shorter treatment duration for the first episode of pneumonia and shorter duration of mechanical ventilation or length of stay in ICU [33].

The most frequently used substances for monotherapy of SCI-AP in this study were acyl-aminopenicillins/β-lactamase inhibitors, cephalosporins group 3, and fluoroquinolones. This is in line with the recommendations made by recent guidelines for the management of HAP in individuals without an increased risk of multidrug-resistant (MDR) pathogens in Germany [34]. The alternative use of these agents in many departments can be interpreted under consideration of local pathogen and resistance profiles. Combination therapy, which should be preserved for individuals at high risk for MDR pathogens [34], was frequently applied in 54% of the departments. This can be explained by an approximately 50% rate of mechanical ventilation in individuals with acute SCI-AP [5]. A prophylactic systemic administration of antibiotics for preventing pneumonia was not applied by 90% of the responding departments. Antibiotics for the prevention of SCI-AP are not recommended in Germany, as the evidence for such prophylactic treatment is still inconsistent [12, 35–37]. Two large clinical trials investigating prophylactic antibiotic treatment in more than 3500 individuals after acute stroke [38, 39] demonstrated a reduced rate of urinary infections but neither prevented stroke-associated pneumonia nor improved stroke outcome. In addition, there is a basic risk of colonization with MDR pathogens or overgrowth infection with Clostridium difficile [38, 39], although it rarely occurred after preventive antibiotic treatment after stroke and was not limited to the treatment arm [38].

A limitation of the chosen study type is that for feasibility reasons the number of vignettes is restricted, and so is the variability of CDC-criteria. Therefore, it is inevitable that CDC-criteria are overlapping in some case vignettes with demographic or injury characteristics of the SCI cases (e.g. fever with AIS A). Nevertheless, the primary aim of this study was to evaluate the utility of CDC-criteria rather than the significance of the individual’s baseline characteristics in diagnosing SCI-AP. Similar to other studies [18, 19], the 38% response rate of this paper-based survey is not meeting the 70% benchmark for mail surveys [40]. This can be partly explained by underestimated response rates due to the fact that addressees, for which the survey is not applicable (e.g. not treating SCI cases in sufficient numbers), are less likely to reply [40]. A lack of representativeness of the survey seems unlikely, because 24 of the 57 responding departments are treating 10–40 cases of SCI and 19 departments more than 40 cases per year, indicating a high-level of SCI-specific expertise among respondents. Given an annual incidence of 36 SCI cases per million inhabitants in Germany [41], the responding departments would cover at least one-third of the acute SCI-population. Nevertheless, because this study reflects the situation in Germany, a re-evaluation of diagnostic approaches for SCI-AP, warranted for the development of tailor-made SCI-specific guidelines, should include international studies on the applicability of CDC-criteria for SCI-AP.

Providing an overview on the current status of diagnostic and treatment algorithms for SCI-AP, the survey’s main conclusions are: (I) X-ray findings provide important guidance for diagnosis of SCI-AP; (II) biomarkers such as C-reactive protein and procalcitonin are frequently requested supplementary parameters; (III) CDC-criteria comprise important items for defining HAP but modifications regarding their clinical feasibility for SCI-AP are required; and (IV) existing centre-specific SOPs in Germany seem to have no substantial influence on diagnostic accuracy of SCI-AP. International guidelines for the diagnosis of SCI-AP, factoring-in SCI-specific parameters of the individual susceptibility for neurogenic immune depression (SCI-IDS), are needed to develop and compare future advances to reduce SCI-AP.

### Data archiving

All data reported in this study are archived in the Department of Experimental Neurology, Charité-Universitätsmedizin Berlin, Berlin, Germany for at least 10 years after publication of the study.

## Supplementary information

Supplementary Appendix
