# Supplementary material for: Clinical decision-making on spinal cord injury-associated pneumonia: a nationwide survey in Germany
Source: Spinal Cord. 2020 Feb 18;58(8):873–81. doi: 10.1038/s41393-020-0435-5 (PMC7223654; doi:10.1038/s41393-020-0435-5)
Supplement: Supplementary file 1 — Supplementary Appendix [file 41393_2020_435_MOESM1_ESM.pdf]

1. General

I. How many patients with spinal cord injuries (SCI) do you treat annually?

☐ none ☐ <10 ☐ 11 – 40 ☐ >40

II. How many beds does your ICU have?

☐ <10 ☐ >10

III. In your assessment, what are the most common complications occurring after SCI?

system: ☐ cardiac ☐ pulmonary ☐ gastro-intestinal ☐ urological ☐ other

origin: ☐ infectious ☐ trauma – associated ☐ other

IV. Are there standardized procedures (SOP) in your clinic for the diagnosis and therapy of pneumonia?

☐ yes ☐ no

If yes, are they available in a document?

☐ yes ☐ no

2. Example cases

The following cases were designed to establish criteria in diagnosis and therapy of pneumonia in patients with acute SCI.

I. On your ICU, you are examining a 30-year old patient, mechanically ventilated after a complete SCI (AIS A) neurological level C4 due to a motorcycle accident. Because of a vertebral body C4 burst fracture, the patient received 3 days ago a ventrodorsal stabilization with decompression. The nurses report increased suctioning of putrid secretion as well as an increased ventilatory demand with increased respiratory frequency. The clinical examination shows inconspicuous auscultation findings, a temperature increase to 38.8°C. Leukocytosis does not exist. In the recent X-ray image newly occurring basal consolidation can be detected.

a. In your expertise, does the patient have a pneumonia? ☐ yes ☐ no

b. Would you start antibiotic treatment? ☐ yes ☐ no

c. Do you need further examinations, to diagnose pneumonia? ☐ yes ☐ no
If yes, which diagnostics? *(multiple choices possible!)*

elevated C-reactive protein ☐

elevated procalcitonin ☐

proof in microbiological probes ☐
(e.g. sputum/tracheal secret/blood cultures)

other:
(please specify)

II. A 73-year-old woman is presented to you at your Intermediate Care Unit 5 days after surgery. The patient fell down a staircase at home and suffered a Th7 fracture with incomplete paraplegia (AIS B). On the day of admission, the fracture was decompressed and dorsally stabilized. During the clinical examination the patient appears confused. She is unable to give any information on temporal and spatial orientation. There is no increase in temperature and no leukocytosis. The blood gas analysis shows a reduced oxygen saturation. In the clinical examination the patient presents a pathological auscultation finding and an indication of a newly occurring cough with progressive secretion. The x-ray image made today shows a suspected basal infiltrate of the left lung.

a. In your expertise, does the patient have a pneumonia? ☐ yes ☐ no

b. Would you start antibiotic treatment at this time? ☐ yes ☐ no

c. Do you need further examinations to diagnose pneumonia? ☐ yes ☐ no
If yes, which diagnostics? *(multiple choices possible!)*

elevated C-reactive protein ☐

elevated Procalcitonin ☐

pathogen detection in microbiological samples ☐
(e.g. sputum/tracheal secret/blood cultures)

other:
(please specify)

III. Two days after surgical treatment of a fracture in the cervicothoracic region with SCI AIS B and concomitant blunt chest trauma, a 42-year-old ventilated patient develops fever of up to 38.1 °C. In the routine laboratory of the intensive care unit, leukocytosis is detected with 13.000/µl. The currently prepared chest x-ray of the patient does not show any pathological findings. Nurses report no change in pulmonary secretion or need of ventilatory assistance, however, the patient seems to suffer from dyspnea. There are no pathological pulmonary auscultatory findings.

a. In your expertise, does the patient have a pneumonia? ☐ yes ☐ no

b. Would you start antibiotic treatment at this time? ☐ yes ☐ no

c. Do you need further examinations to diagnose pneumonia? ☐ yes ☐ no
If yes, which diagnostics? *(multiple choices possible!)*

| **elevated C-reactive protein** ☐

| **elevated Procalcitonin** ☐

| **pathogen detection in microbiological samples** ☐
(e.g. sputum/tracheal secret/blood cultures)

| **other:**
(please specify)

IV. An 80-year-old patient, who is in intensive care on the 3rd day after surgical stabilization and decompression after complete thoracic SCI (AIS A) develops a newly occurring cough with sputum. The patient, who is well oriented in time and place, shows no pathological findings in the auscultation except for tachypnea (32/min). The routine laboratory is normal, as is the blood gas analysis, there is no temperature increase. In the present chest X-ray image, as in the initial image, small, planar infiltrates are visible. The following pre-existing conditions are known from the medical history: Diabetes mellitus type II, arterial hypertension, COPD.

a. In your expertise, does the patient have a pneumonia? ☐ yes ☐ no

b. Would you start antibiotic treatment at this time? ☐ yes ☐ no

c. Do you need further examinations to diagnose pneumonia? ☐ yes ☐ no
If yes, which diagnostics? *(multiple choices possible!)*

| **elevated C-reactive protein** ☐

| **elevated Procalcitonin** ☐

| **pathogen detection in microbiological samples** ☐
(e.g. sputum/tracheal secret/blood cultures)

| **other:**
(please specify)

V. On your daily routine on your intensive care unit, the chest-x ray of a 75-year old female patient with incomplete brachial-accentuated spinal cord injury (AIS D) due to fracture of C6/7 is presented. There is radiological evidence of new bilateral infiltrates. In clinical inspection, the demented patient appears to be still disoriented and confused 4 days after operation with new cough without secretion. Pulmonary auscultation reveals silent fine crackles. Laboratory chemistry, however, shows no abnormalities, fever is not detectable, pulse oximetry shows no saturation deficit.

a. In your expertise, does the patient have pneumonia? ☐ yes ☐ no

b. Would you start antibiotic treatment at this time? ☐ yes ☐ no

c. Do you need further examinations to diagnose a pneumonia? ☐ yes ☐ no
If yes, which diagnostics? *(multiple choices possible!)*

| **elevated C-reactive protein** ☐

| **elevated Procalcitonin** ☐

| **pathogen detection in microbiological samples** ☐
(e.g. sputum/tracheal secret/blood cultures)

| **other:**
(please specify)

VI. A 45-year old construction worker with incomplete SCI (AIS C) neurological level L2 due to a L2-vertebral fracture after an occupational accident underwent stabilization and decompression surgery 7 days ago. He has a newly elevated body temperature of 39.2°C since this morning time. Clinical examination shows increased respiratory rate of 28/min, however, auscultatory finding appear normal, there is no cough or secretion. The blood gas analysis carried out shows discreetly worsened parameters. In the blood cell count, a leukocytosis is conspicuous. The present CT scan shows a normal result.

a. In your expertise, does the patient have a pneumonia? ☐ yes ☐ no

b. Would you start antibiotic treatment at this time? ☐ yes ☐ no

c. Do you need further examinations to diagnose pneumonia? ☐ yes ☐ no
If yes, which diagnostics? *(multiple choices possible!)*

| **elevated C-reactive protein** ☐

| **elevated Procalcitonin** ☐

| **pathogen detection in microbiological samples** ☐
(e.g. sputum/tracheal secret/blood cultures)

| **other:**
(please specify)

VII. Due to a new progredient secretion, you perform a bronchoscopy in an intubated 63-year old female patient who underwent surgery of a thoracolumbar vertebral fracture with incomplete SCI (AIS C). You are able to suction putrid secretions. A leukocytosis could be excluded in the laboratory. The blood gas analysis performed showed no pathological findings. There is no fever, the auscultation appears inconspicuous with unchanged respiratory behavior, the current X-ray shows two single soft opacities.

a. In your expertise, does the patient have a pneumonia? ☐ yes ☐ no

b. Would you start antibiotic treatment at this time? ☐ yes ☐ no

c. Do you need further examinations to diagnose pneumonia? ☐ yes ☐ no
If yes, which diagnostics? *(multiple choices possible!)*

| **elevated C-reactive protein** ☐

| **elevated Procalcitonin** ☐

| **pathogen detection in microbiological samples** ☐
(e.g. sputum/tracheal secret/blood cultures)

| **other:**
(please specify

VIII. In your daily radiological conference on your intensive care unit, chest x-ray of a 24-year old male patient with SCI AIS D at level C4 after a motorcycle accident with concurring blunt thoracic and abdominal trauma is presented. Consolidations of the basal lung segments on the right side are shown. On request, leukopenia and increased ventilator requirements with increased secretion of purulent sputum are confirmed. Fever and signs of increased respiratory rate and coughing are denied. The auscultation did not reveal anything.

a. In your expertise, does the patient have a pneumonia? ☐ yes ☐ no

b. Would you start antibiotic treatment at this time? ☐ yes ☐ no

c. Do you need further examinations to diagnose pneumonia? ☐ yes ☐ no
If yes, which diagnostics? *(multiple choices possible!)*

| **elevated C-reactive protein** ☐

| **elevated Procalcitonin** ☐

| **pathogen detection in microbiological samples** ☐
(e.g. sputum/tracheal secret/blood cultures)

| **other:**
(please specify)

3. Therapeutic procedures

This part is about therapy schemes, substance classes and management algorithms.

I. How do you administer the initial therapy? ☐ orally ☐ systemically

IIa. Which groups of antibiotics do you administer as part of an initial therapy for pneumonia in a patient with spinal cord injury?

| A | **Aminopenicillins/β-Lactamase-Inhibitors** ☐
(e.g. Ampicillin/Sulbactam)

| B | **Acylaminopenicillins/β-Lactamase-Inhibitors** ☐
(e.g. Piperacillin/Tazobactam)

| C | **Carbapenems** ☐
(e.g. Imipenem, Meronem)

| D | **Cephalosporines Group 2** ☐
(e.g. Cefuroxim)

| E | **Cephalosporines Group 3** ☐
(e.g. Ceftriaxon)

| F | **Fluorquinolones** ☐
(e.g. Moxifloxacin)

| G | **Macrolides** ☐
(e.g. Clarythromycin)

| H | **Others:** ☐
(please specify)

b. Do you administer combination of above-mentioned substances ☐ yes ☐ no

If yes, please specify?
(please mark corresponding letters (see above))

**substance**	**A**	**B**	**C**	**D**	**E**	**F**	**G**	**H**
**with substance**	**A**	**B**	**C**	**D**	**E**	**F**	**G**	**H**

IIIa. Do you adhere to a time limit for administration of the substance? ☐ yes ☐ no

If yes, how long is therapy administered? ☐ ________ days

b. Do you monitor therapeutic success? ☐ yes ☐ no

If yes, please specify? *(multiple choices possible!)*

- **clinical examination** ☐
  (e.g. auscultatory findings)
- **blood-tests** ☐
  (e.g. CRP, pCT, BGA)
- **radiological** ☐
  (e.g. x-ray)

If therapy success is monitored, when after the start of therapy is it first assessed? _______ hours

c. Are decisions on for further treatment based on antibiograms? ☐ yes ☐ no

IIIa. Do patients with spinal cord injury receive prophylactic antibiotic treatment? ☐ yes ☐ no

If yes, who receives prophylactic treatment?

- **all** ☐
- **patients with certain severity** ☐ _________
  (please specify)
- **patients with dysphagia** ☐
- **patients fulfilling other criteria** ☐
  (please specify)

b. Do you perform respiratory therapy? ☐ yes ☐ no

If yes, when does it start? ☐ from_________

c. Do you perform dysphagia and aspiration prophylaxis? ☐ yes ☐ no

If yes, when does it start? ☐ from_________

d. Are you familiar with the so-called CDC-criteria? ☐ yes ☐ no

If yes, what is your personal evaluation of their clinical relevance? ☐ high ☐ moderate ☐ irrelevant
